# Structural Diversity of the Active N-Terminal Kinase Domain of p90 Ribosomal S6 Kinase 2

**DOI:** 10.1371/journal.pone.0008044

**Published:** 2009-11-30

**Authors:** Margarita Malakhova, Igor Kurinov, Kangdong Liu, Duo Zheng, Igor D'Angelo, Jung-Hyun Shim, Valerie Steinman, Ann M. Bode, Zigang Dong

**Affiliations:** 1 Department of Cellular and Molecular Biology, The Hormel Institute, University of Minnesota, Austin, Minnesota, United States of America; 2 Northeastern Collaborative Access Team (NE-CAT), Advanced Photon Source (APS), Cornell University, Argonne, Illinois, United States of America; 3 Canadian Macromolecular Crystallography Facility, Canadian Light Source Inc., Saskatoon, Canada; 4 Biochemistry Department, College of Saint Benedict, St. Joseph, Minnesota, United States of America; University of Washington, United States of America

## Abstract

The p90 ribosomal protein kinase 2 (RSK2) is a highly expressed Ser/Thr kinase activated by growth factors and is involved in cancer cell proliferation and tumor promoter-induced cell transformation. RSK2 possesses two non-identical kinase domains, and the structure of its N-terminal domain (NTD), which is responsible for phosphorylation of a variety of substrates, is unknown. The crystal structure of the NTD RSK2 was determined at 1.8 Å resolution in complex with AMP-PNP. The N-terminal kinase domain adopted a unique active conformation showing a significant structural diversity of the kinase domain compared to other kinases. The NTD RSK2 possesses a three-stranded βB-sheet inserted in the N-terminal lobe, resulting in displacement of the αC-helix and disruption of the Lys-Glu interaction, classifying the kinase conformation as inactive. The purified protein was phosphorylated at Ser227 in the T-activation loop and exhibited *in vitro* kinase activity. A key characteristic is the appearance of a new contact between Lys216 (βB-sheet) and the β-phosphate of AMP-PNP. Mutation of this lysine to alanine impaired both NTDs *in vitro* and full length RSK2 *ex vivo* activity, emphasizing the importance of this interaction. Even though the N-terminal lobe undergoes structural re-arrangement, it possesses an intact hydrophobic groove formed between the αC-helix, the β4-strand, and the βB-sheet junction, which is occupied by the N-terminal tail. The presence of a unique βB-sheet insert in the N-lobe suggests a different type of activation mechanism for RSK2.

## Introduction

The 90-kDa ribosomal S6 kinase 2 (RSK2) is a serine-threonine kinase, which plays a key role in the mitogen-activated protein (MAP) kinase signaling pathway. RSK2 is activated in response to a variety of stimuli, including insulin, growth factors, neurotransmitters, and chemokines [1]. The RSK2 pathway is a key regulator of cancer cell proliferation [2]–[4]. RSK2 phosphorylates a variety of substrates, including the cAMP-responsible element-binding 2 protein [5], histone H3 [6], the tumor suppressor p53 protein [7], cell cycle proteins Myt1 [8] and Bub1 [9], transcriptional factors including c-Fos [10], [11], activating transcriptional factor-4 [12], and nuclear factor of activated T cell [13].

In mammals, the RSK family comprises four closely related isoforms, RSK1-4. RSK and MSK (mitogen- and stress-activated protein kinase) constitute a family of protein kinases that mediate signal transduction downstream of the MAP kinase cascades. Among the many serine-threonine kinases, only two unique kinases, RSK and MSK, contain two distinct kinase domains in a single polypeptide chain, separated by a linker region of ∼100 amino acids [14]. Both domains are catalytically functional [15], and the C-terminal kinase domain (CTD) regulates the activity of the N-terminal domain (NTD) through phosphorylation of the hydrophobic linker region. The current mechanism of activation of full length RSK2 is suggested to occur through the activation of the C-terminal protein kinase domain by its interaction with and phosphorylation by ERK1/2 [16]–[18]. The activated CTD RSK2 autophosphorylates Ser386 in the linker region [19], which becomes a docking site for PDK1 [20] that activates the NTD RSK2 through phosphorylation of Ser227 in the T-activation loop [21].

The crystal structure of the C-terminal protein kinase domain (CTD) RSK2 was recently solved in our laboratory [22]. The structure of the RSK2 full length protein or the separate RSK2 N-terminal domain, which is responsible for phosphorylation of different endogenous substrates, is still unknown. The crystal structure of the N-terminal domain of a close homologue, MSK1, was reported in its inactive conformation with a distorted nucleotide binding loop that occluded the ATP binding site [23]. The authors suggested that an inactive MSK1 NTD conformation is stabilized by a newly formed three-stranded β-sheet in the N-terminal lobe. The activation mechanism of the MSK1 NTD was proposed to occur through a transition from the βB-sheet insert to the αB-helix.

The recently published NTD RSK1 structure (residues 56–340) showed a disordered N-terminal end, T-activation loop, and αC-helix [24]. Although the NTD RSK1 was shown to form a complex with an ATP analogue and two inhibitors, staurosporine and purvalanol A, the authors indicated that the structural conformation of RSK1 was inactive because the ATP analogue was bound in an orientation not favorable for phospho-transfer and the protein was also catalytically inactive in solution.

The NTD RSK2 is thought to belong to the growth factor-activated AGC superfamily of kinases (protein kinase A/protein kinase G/protein kinase C), which have the kinase domain followed by the C-terminal hydrophobic motif (HM) tail. Phosphorylation of the hydrophobic motif residues in combination with phosphorylation of the T-loop results in the synergistic activation of AGC kinases [25]–[28]. Among others, the AGC family includes the cAMP-dependent protein kinase (PKA), Akt (PKB), protein kinase C (PKC), and 3-phosphoinositide-dependent protein kinase-1 (PDK1). The nonphosphorylated, inactive PKB possesses a completely disordered αC-helix [26]. A structural study of the activated PKB showed that the phosphorylated HM occupies a hydrophobic motif pocket formed at the N-terminal lobe, leading to the ordering of the αC-helix and stabilization of the kinase active conformation [29]. PDK1, the master regulator of AGC kinase signal transduction, activates its substrates by phosphorylation of the T-activation loop. PDK1 is an atypical AGC member because it does not possess the characteristic hydrophobic C-terminal tail. The region of PDK1 that interacts with the phosphorylated HM of its substrates is located at the same pocket at the N-lobe of the catalytic domain [30]–[32]. Neither the N-terminal kinase domain of the MSK1 or RSK1 structure showed localization of the C-terminal hydrophobic tail or the presence of the αB-helix, a secondary element that is usually located in the N-lobe of AGC-kinases. The absence of the C-terminal extension in the protein constructs and the absence of the αB-helix in the crystal structures of RSK1 and MSK1 were proposed as additional reasons for the enzyme inactivity [23], [24].

The available physiological data imply that the N-terminal protein kinase domain of both RSK and MSK is involved in the phosphorylation of a variety of substrates, and is believed to have an active kinase conformation. Unexpectedly, the crystal structures of MSK1 and RSK1 exhibited the inactive conformation. Herein, we present an analysis of the refined high-resolution structure of the isolated recombinant N-terminal protein kinase domain (NTD) of RSK2 in the active conformation complexed with an ATP analogue. The kinase activity was confirmed by a complimentary enzyme assay.

## Results and Discussion

### Overall Structure

The X-ray crystal structure of the N-terminal kinase domain of RSK2 (residues 44–367) complexed with AMP-PNP was refined to 1.8 Å resolution. The structure was solved by molecular replacement using the available NTD MSK1 (residues 24–345) structure as a search model (PDB code 1VZO). At the time of our structure determination, the coordinates for NTD RSK1, which are now available (PDB code 2Z7Q) in the protein data bank, were not yet released. The final RSK2 model has an R-factor equal to 20.4% and R-free equal to 24.8% with good stereochemistry. The unit cell parameters, details of data collection, and refinement statistics are presented in Table 1.

**Table 1:** X-ray data collection and refinement statisticsa).

Data collection	
Space group	P2_1_2_1_2_1_
Cell dimensions	
*a*, *b*, *c* (Å)	51.17, 51.38, 140.87
Resolution b) (Å)	36-1.8 (1.86-1.80)
*R*_merge_	0.091 (0.89)
R_p.i.m._^c^	0.041 (0.39)
*I*/σ	15.2 (2.0)
Completeness (%)	99.4 (94.8)
Redundancy	6.2 (5.0)
Refinement	
Resolution (Å)	36-1.8
No. reflections	32826
*R*_work/_ *R*_free_^d^	0.204/0.248
No. atoms	
Protein	2518
AMP-PNP	31
Water	274
B-factors (Å^2^)	
Protein	31.0
AMP-PNP	26.3
Water	35.6
R.m.s. deviations	
Bond lengths (Å)	∼0.003 Å
Bond angles (°)	∼0.6 Å

a) One crystal was used for the structure determination.

b) Highest resolution shell is shown in parenthesis.

c) Precision-indicating merging R factor.

d) R_free_ was calculated from a randomly chosen 5% of reflections excluded from refinement.

**Figure 1:**
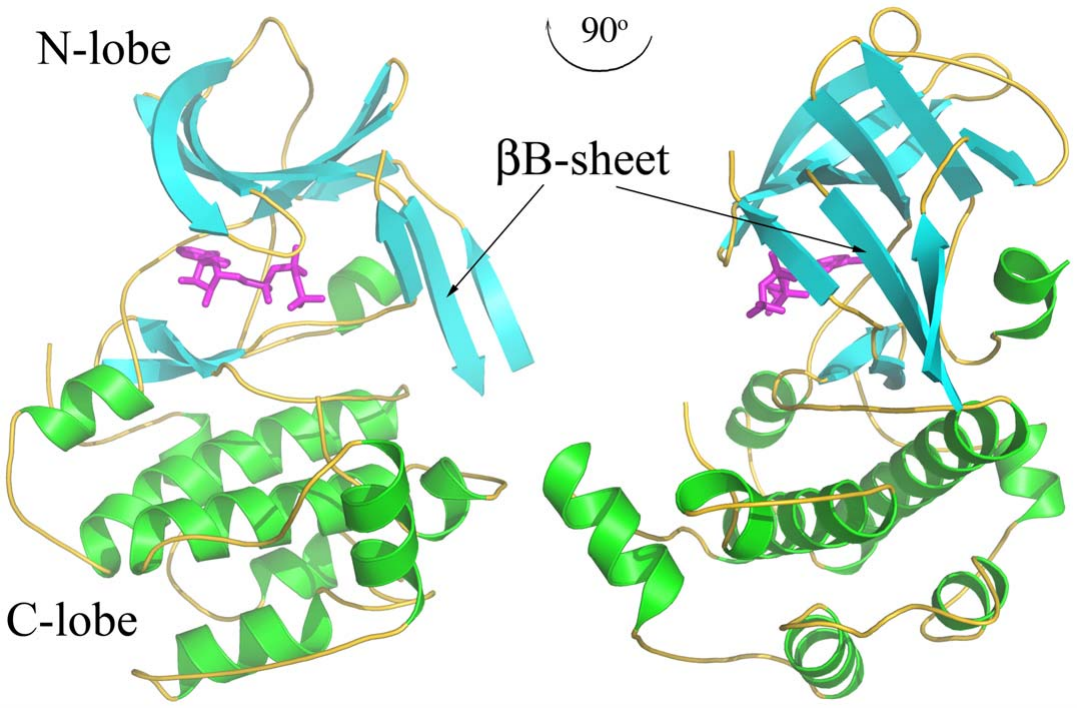
A ribbon diagram of the NTD RSK with two views rotated by 90°. The β-strands are shown in green, the α-helices are in cyan, and the coils are shown in yellow. The AMP-PNP molecule is shown in sticks presentation and colored magenta. The new βB-sheet insert is indicated by the arrow.

The NTD RSK2 adopted an overall protein kinase two-lobal scaffold with the ATP-binding site in the cleft between the small N-terminal lobe and large α-helical C-terminal lobe (Figure 1). The protein was concentrated and crystallized in the presence of a non-hydrolyzable ATP analogue, AMP-PNP, which was easily recognized in the electron density map inside the active site. The T-activation loop (residues 220–230), part of the αC-helix (residues 111–119), and the N- and C-termini (residues 44–46 and 347–367, respectively) were not visible in the electron density map and were not included in the current model.

**Figure 2:**
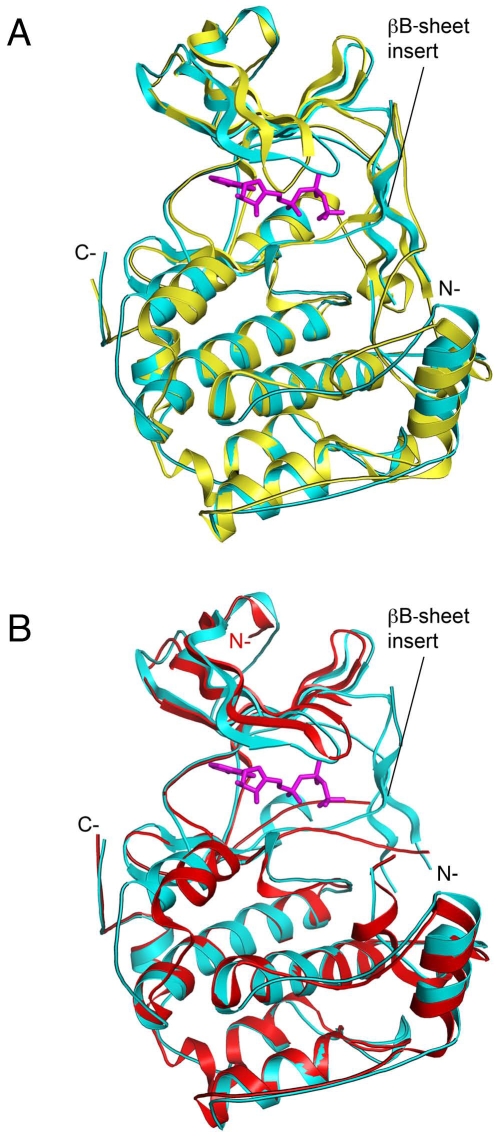
Superimposition of the N-terminal kinase domain of RSK2 with the similar domain of MSK1 (A) or RSK1 (B). The NTD RSK2 is shown in cyan in both (A) and (B). The inactive NTD MSK1 (PDB code 1VZO) is shown in yellow (**A**) and the inactive NTD RSK1 (PDB code 2Z7Q) is shown in red (**B**). The ATP molecule from the RSK1 structure is omitted. Obviously, the disordered region in RSK1 may accommodate the βB-sheet.

The refined NTD RSK2 structure showed an overall similar structure with NTD MSK1, including an unusual βB-sheet insert in the N-terminal lobe (Figure 1, 2A). Two kinase structures are superimposed with a root mean square deviation (r.m.s.d.) of 1.46 Å for the corresponding C_α_ atoms. The available crystal structure of NTD RSK1 did not provide any insight as to whether the βB-sheet insert exists in the N-lobe, because the purported βB-sheet residues are mostly located in the disordered region of the RSK1 structure. A two-structure superimposition showed that the N-lobe of RSK1 has enough room for the βB-sheet to be inserted without too many steric clashes (Figure 2B).

### NTD RSK2 Is Active In Vitro

**Figure 3:**
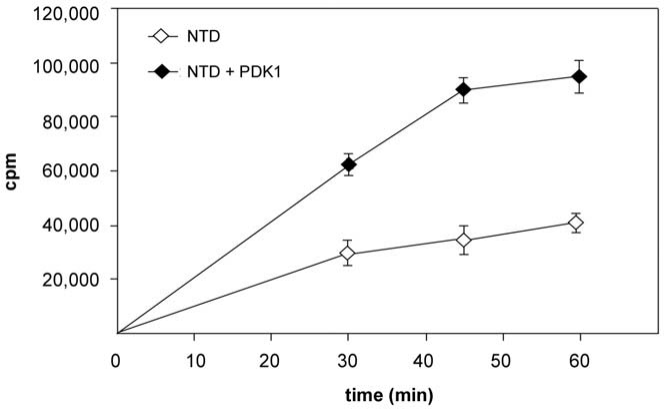
The NTD RSK2 exhibits intrinsic kinase activity. The protein was incubated with a biotinylated S6 peptide and [γ-^32^P] ATP for 60 min at 30°C. Where indicated, the protein was pre-activated by active PDK1 for 10 min. The radiolabeled, phosphorylated S6 peptide was recovered from the reaction mixture by biotin capture membrane. The contribution of PDK1 to S6 peptide was negligible.

We were interested to determine whether the isolated NTD RSK2 protein had kinase activity. The NTD RSK2 with the truncated hydrophobic linker (residues 1–360) was phosphorylated by PDK1 at Ser 227 in the T-activation loop, which resulted in increased activity toward the S6 peptide *ex vivo* and *in vitro* [21]. Taking advantage of this fact, we performed an *in vitro* kinase assay using a purified His-fusion NTD RSK2 (residues 44–367) with and without activation by PDK1 and a biotinylated S6 peptide. Surprisingly, the isolated NTD RSK2 displayed *in vitro* kinase activity toward the S6 peptide even without activation by PDK1 (Figure 3). To determine why NTD is active, we performed Western blot analysis that showed that Ser 227 was phosphorylated in the purified protein (Figure S1). Pre-incubation of NTD with active PDK1 for 10–20 min increased the enzyme activity 1.5 – 3 fold most likely because of additional phosphorylation of the partially phosphorylated NTD fragment. The crystallized protein was also phosphorylated at Ser 227 as shown by Western blot. The T-activation loop region, including that serine residue, was disordered in the current structure.

### The ATP-Binding Site

**Figure 4:**
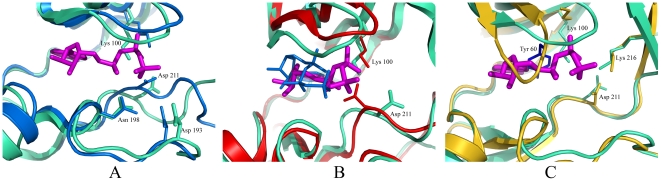
A comparison between the active sites of NTD RSK2 and active PKA (A), inactive NTD RSK1 (B), and inactive NTD MSK1 (C). RSK2 is shown in cyan in all panels, and only the residues of RSK2 are labeled. The active site cleft is shown with approximately the same view and zoom. (**A**) Active PKA (PDB code 1ATP) is shown in blue. The AMP-PNP molecule (magenta sticks) is depicted only from the RSK2 structure and is omitted from the PKA structure for simplification (because of a nearly overlapping conformation). The positions of most of the conserved active site residues of RSK2 are similar to those of active PKA. Those residues are Lys100 of RSK2 and Lys72^PKA^ located on the β3-strand; Asn198 and Asn171^PKA^ from the catalytic loop; Asp211 (DFG-motif) and Asp184^PKA^; and Asp193 (RD-motif) and Asp166^PKA^. (**B**) The inactive NTD RSK1 (PDB code 2Z7Q) is shown in red with the bound ATP molecule shown in blue. The overall view is rotated by ∼10° about the vertical axis relative to panels (A) and (C) to show the difference in orientation of the ribose ring and phosphate groups. Lys94^RSK1^, an analogue of the Lys100^RSK2^ residue, does not interact with the phosphate group and forms a hydrogen bond with the Asp205 from the DFG-motif. Asp205^RSK1^ occupies a different position compared with Asp211^RSK2^. (**C**) The inactive NTD MSK1 (PDB code 1VZO) is shown in yellow. The position of the glycine-rich loop (P-loop) is distorted in the MSK1 structure. As a result, Tyr60^MSK1^ (blue) occludes the binding of the ATP molecule. A lysine from the β3-strand, Lys81^MSK1^, is oriented perpendicularly compared with Lys100^RSK2^ and forms an H-bond with the P-loop. Lys216^RSK2^ and Lys200^MSK1^ from the β9-strand occupy the same position.

The AMP-PNP molecule was modeled into a well-defined electron density in the NTD RSK2 active site. The omit electron density map showed a continuous electron density perfectly covering the adenine, sugar, and phosphate groups. The detailed interaction of AMP-PNP with the active site amino acid residues is presented in Figure S2, which was generated by *Ligplot* [33]. We checked the position of all the conserved residues in the ATP-binding pocket and compared them with corresponding residues in active PKA and inactive RSK1 or MSK1. The superimposition of the ATP-binding site of the NTD RSK2 and active PKA is illustrated in Figure 4A. The overall hydrogen bonding network between the AMP-PNP molecule and invariant amino acid residues in the RSK2 catalytic site is similar to that of PKA, which is optimal for phosphotransfer [34]–[37]. The conserved Lys100 from the β3-strand, a crucial residue responsible for correct alignment of ATP phosphates for catalysis, coordinates α- and β-phosphate groups and occupies a position similar to Lys72^PKA^. Another conserved residue, Asn198 of NTD RSK2 (analogue Asn171^PKA^), located on the catalytic loop, forms two hydrogen bonds with α- and γ-phosphates (distance 2.79 Å and 3.11 Å, respectively). All other conserved residues from the DFG-motif (residues 211–213) and the RD-motif (residues 192–193) have a similar conformation to the corresponding residues of PKA, as clearly seen in Figure 4A.

When comparing the RSK2 structure with inactive RSK1 and MSK1, the positions of some of the conserved residues are different (Figure 4B and 4C). The catalytically inactive NTD RSK1 forms a nonproductive complex with ATP as was described previously [24]. The conformational position of the three phosphate groups and ribose ring moiety was not aligned properly for phosphotransfer (Figure 4B). Displacement of the ATP molecule from its proper position does not allow the conserved Lys94^RSK1^ from the β3-strand (analogue of Lys100^RSK2^) to coordinate the phosphate groups in the RSK1 structure. Instead, Lys94^RSK1^ forms a hydrogen bond with an aspartate (DFG-motif), a position that differs from Asp211^RSK2^.

The conformation of the phosphate-binding loop in MSK1 prevents the binding of ATP as was described [23] and demonstrated in Figure 4C. The side chain of Tyr60^MSK1^ occludes the adenosine moiety binding. Deformation of the P-loop most likely resulted in the misalignment of a crucial Lys81 MSK1 from the β3-strand. Lys81^MSK1^ is rotated ∼90° compared to the analogue Lys100^RSK2^ and forms a hydrogen bond with the β2-strand of the P-loop. We conclude that the distortion of the phosphate-binding loop and consequent blockage of the ATP binding site by the side chain of Tyr60 is a primary reason for MSK1 inactivity. Superimposition of the active sites of the N-terminal domains of RSK2 and MSK1 clearly showed no deformation of the P-loop in RSK2, which is arranged properly allowing a productive binding of ATP in the active site pocket. The conformation of all other conserved residues from the DFG-motif (residues 211–213), RD-motif (residues 192–193), and catalytic Asp198 in the RSK2 structure closely resembles that observed in MSK1.

### Novel β-Sheet Insert in the N-Lobe

**Figure 5:**
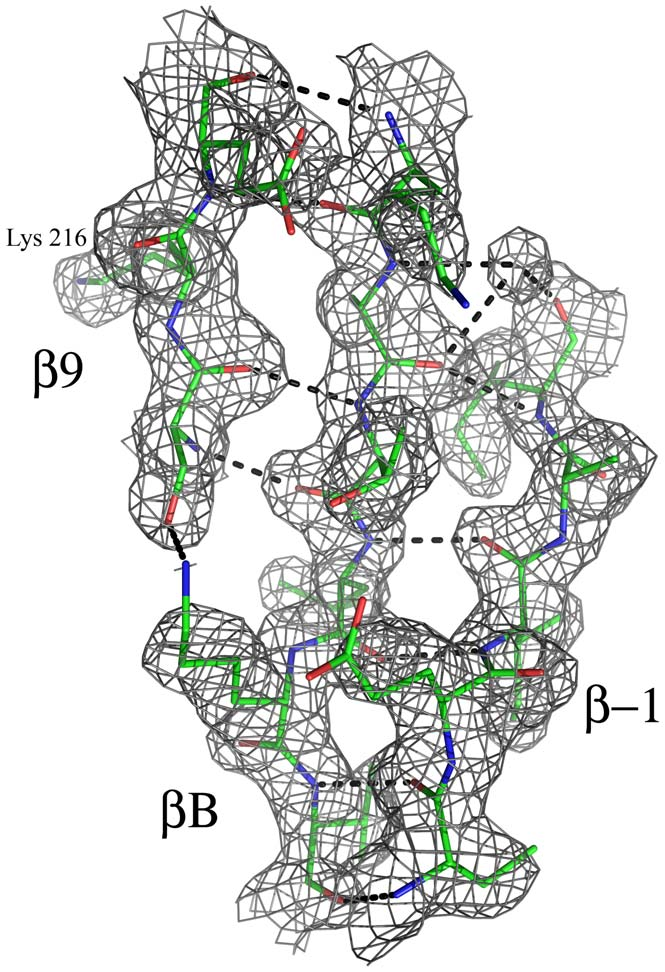
The electron density from the final 2Fo-Fc map for the βB-sheet insert. The βB-sheet insert consists of three β-strands: β-1, βB, and β9-strands, which are held together by the hydrogen bonding network. The hydrogen bonds between the strands are shown as dotted lines. Oxygen atoms are in shown in red, nitrogen atoms are in blue, and carbons in green. The lysine 216 residue located on the βB-strand is labeled.

The N-lobe of the NTD RSK2, traditionally composed of twisted, five stranded β-sheets, has a new intriguing feature. The novel βB-sheet, unusual for most kinases, was inserted into the N-lobe (Figure 1). The βB-sheet insert is comprised of three antiparallel β-strands: the β-1-strand (residues 48–52) at the N-terminal end; the novel βB-strand (residues 104–109); and the β9-strand of the activation segment (residues 215–217). Within the N-lobe, three β-strands were well defined in the electron density map (Figure 5). Threaded together by the hydrogen bond network, three β-strands comprise the stable βB-sheet. The N-terminal β-1-strand, which is outside of the protein kinase domain and harmonically embedded in the kinase fold, forms several hydrogen bonds with the middle βB-strand. The β9-strand, an element of the activation segment, is turned upward, and hydrogen bonded with the βB-strand as well.

A similar βB-sheet insertion in the N-terminal kinase lobe was previously observed in the NTD MSK1, and was suggested to stabilize its inactive conformation [23]. Our comparison of the active site of the ATP-bound RSK2 with the non-ATP-bound MSK1 structure (Figure 4C) clearly illustrates that distortion of the correct position of the phosphate-binding loop and consequent occlusion of ATP binding by Tyr60^MSK1^ was not due to insertion of the βB-sheet into the N-lobe. We showed that the presence of the βB-sheet in the NTD RSK2 does not impair the ability of the enzyme to exhibit phosphotransferase activity (Figure 3) or to bind strongly with ATP in the crystal form (Figures 1, 4). Rather, we suggest that a new βB-sheet insert, instead of the αC-helix, stabilizes the N-terminal kinase lobe active conformation. In the NTD RSK2 structure, the αC-helix is pushed away from its regular position by the βB-sheet (Figure 1), and consequently, the conserved Glu118 residue (invisible in the structure) from the αC-helix cannot interact with Lys100. Despite the absence of a salt bridge, the N-lobe maintains its integrity by the appearance of the new βB-sheet.

### Involvement of Lys216 in Phosphate Binding

**Figure 6:**
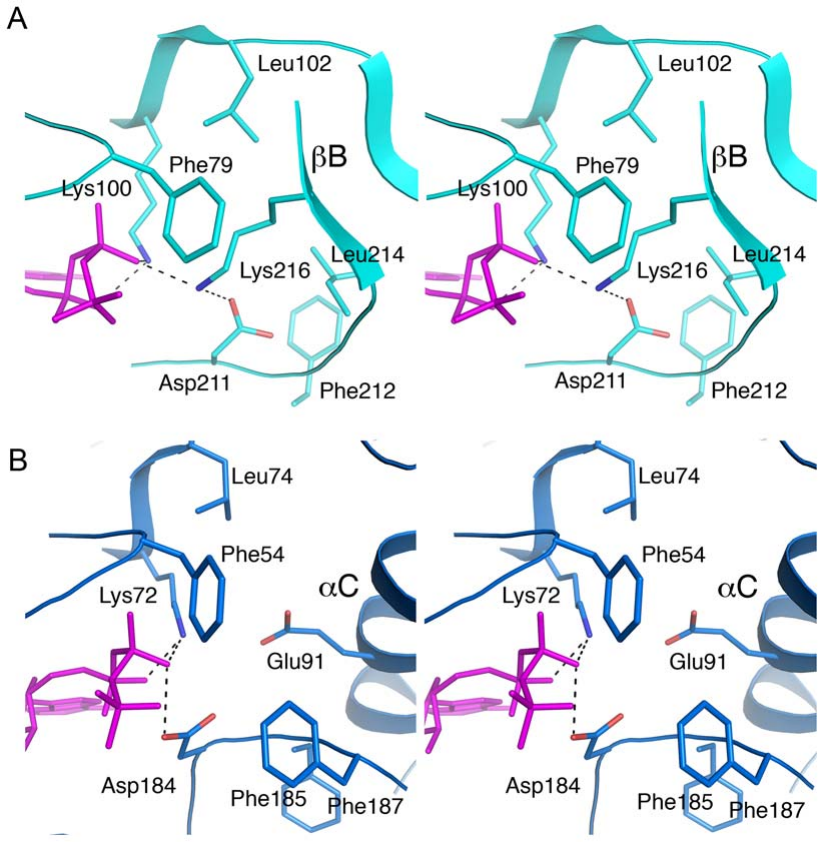
The hydrophobic patch and role of Lys216. The stereo view of the hydrophobic patch formed deep inside the N-lobe and lined by hydrophobic residues is shown for NTD RSK2 in cyan (**A**), and in blue for PKA (PDB code 1ATP) (**B**). The AMP-PNP molecule is shown in magenta. Lys216, located on the β9-strand of the activation segment and interacting with the β-phosphate and Asp211, introduces a charge to the cavity similar to Glu91^PKA^.

Examination of the active site revealed a Lys216 residue engaged in ATP binding (Figures 4C, 6A). The lysine, located on the βB-sheet, interacts with the β-phosphate (distance 2.66 Å) and the conserved Asp211 (distance 2.86 Å) from the DFG-motif. The Lys216 occupies a central position in the deep cavity formed inside the N-lobe. The hydrophobic cavity is lined by Leu214, Leu102, and Phe212 and is shielded by the side chain of Phe79 from the P-loop (Figure 6A). Similarly, in PKA, the cavity is lined by Phe187, Leu74, Phe185, and Phe54 (Figure 6B). The Glu91^PKA^ introduces the charge to that hydrophobic patch and interacts with Lys72^PKA^. The lack of a glutamic acid residue (Glu118 from the αC-helix) in RSK2 due to disruption of the Lys-Glu salt bridge creates a void of charges, and Lys216 compensates by introducing a positive charge to the hydrophobic cavity. A comparison with PKA illustrates that Lys216 may “substitute” for the displaced Glu118 (Glu91^PKA^) residue, even though they have different charges. Instead of the salt bridge, Lys100-Glu118, a new mode of connection appeared – Lys100-β-phosphate-Lys216. The internal volume of the ATP-binding site in the NTD RSK2 is slightly larger (1130 Å) compared with PKA (1115 Å) due to a secondary structure re-arrangement (appearance of the βB-sheet).

### Mutation of Lys216 Supports Its Role

The involvement of one more residue, in addition to Lys100 from the β3-strand, for the coordination of the β-phosphate comprises a novel binding contact never before seen in other kinases complexed with ATP. The corresponding Lys200 residue in the apo form NTD MSK1 occupies a similar conformation (Figure 4C) and contacts an aspartate residue from the DFG-motif as well. However, without an ATP bound in the active site and a distorted phosphate-binding loop, to predict the importance of that lysine residue in MSK1 was difficult.

**Figure 7:**
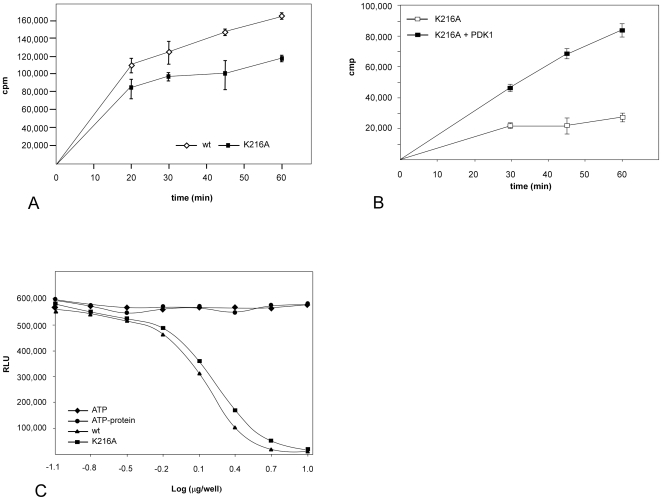
Comparison of wildtype NTD and K216A mutant phosphotransferase activity. (**A**) The K216A mutant showed a reduction of approximately 30% in activity toward the S6 peptide compared with wildtype. A kinetics assay was performed with a biotinylated S6 peptide and [γ-^32^P] ATP as described in Materials and Methods. (**B**) Pre-incubation with active PDK1 for 10 min stimulated K216A kinase activity about two fold in a similar pattern as the wildtype protein. (**C**) Kinase-Glo^®^ luminescent kinase assay. The kinase titration was performed in the presence of ATP and the S6 peptide. Control experiments with ATP alone (no protein, no substrate) or ATP with protein (no substrate) are shown. Neither of proteins showed autophosphorylation activity. The control experiment with only substrate (no protein) showed the same luminescent signal as ATP alone and is not shown in the graph. All experiments were reproduced several times.

To determine whether Lys216 actually participates in phosphate binding or whether its position is a crystal artifact, we mutated Lys216 to alanine to prevent the interaction introduced by Lys216. We expected that the mutation would have an effect on substrate phosphorylation if the Lys216 facilitates ATP binding. An *in vitro* kinase assay using [^32^P]ATP and a biotinylated S6 peptide showed that the K216A mutant exhibited an impaired functionality compared with wildtype at all measured time points (Figure 7A). Stimulation of the mutant K216A NTD with PDK1 enhanced protein activity in a manner similar to wildtype (compare Figure 7B and Figure 3). The Western blot showed an approximate equal level of phosphorylation at Ser227 for the two proteins (Figure S1). We assume that the observed reduction of approximately 30% in activity was a result of the mutation.

To determine whether the wt NTD or K216A mutant might autophosphorylate itself thereby increasing its intrinsic activity, we performed a luminescent kinase assay. The assay measures the amount of ATP remaining in solution following a kinase reaction and allows a comparison of the effective doses for wild type and mutant (Figure 7C). Serial two-fold dilutions of protein were made across a 96-well plate in 50 µl buffer containing the S6 peptide. The kinase reaction was started by addition of ATP, carried out for 60 minutes at room temperature, and terminated by adding the luminescent Kinase-Glo^®^ Reagent. The wildtype and K216A mutant showed a different capability to phosphorylate the S6 peptide and neither exhibited an autophosphorylation activity. The effective concentrations (EC_50_) calculated from the curves for NTD wt and the K216A mutant were 1.4±0.2 µg and 1.9±0.1 µg, respectively. The result shows that a higher amount of mutant is required to reach 50% maximal response. The slightly, but not dramatically, reduced enzyme activity of the K216A mutant indicates that a single point mutation did not introduce global conformational changes into the N-lobe, and confirmed the structural finding that Lys216 participates in phosphate binding.

**Figure 8:**
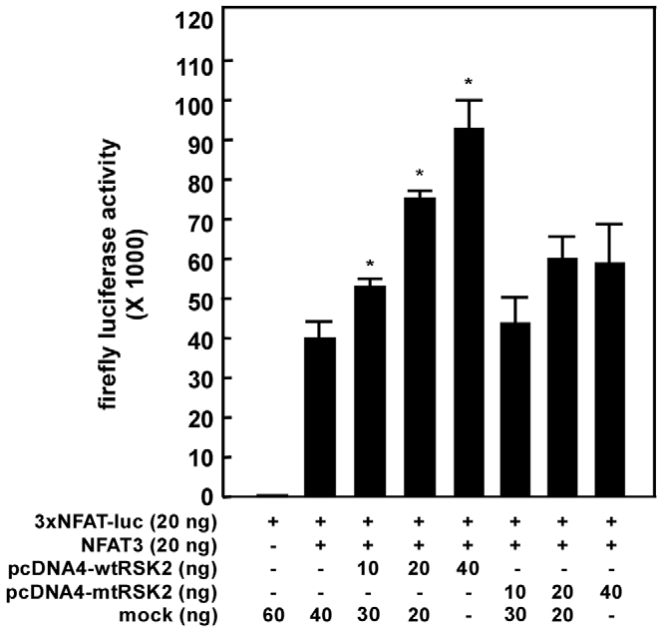
Mutation of Lys216 results in inhibition of full length RSK2 activity *ex vivo*. Wildtype RSK2 induced NFAT3 activity in a dose-dependent manner. K216A mutant showed reduced activity. The 3×NFAT-luciferase reporter plasmid was co-transfected with pcDNA3-Flag-NFAT3, pcDNA4-RSK2, or pcDNA4-RSK2-K216A plasmids into HEK 293 cells. The firefly luciferase activity was analyzed after 36 h and normalized against *Renilla* luciferase activity. Data are presented as the mean±S.D. of values from triplicate experiments. The differences were evaluated using the Student's t-test (*, p<0.05). The equivalent level of each expressed protein was confirmed by Western blot and shown in Figure S3.

To explore the role Lys216 mutation in RSK2 activity *ex vivo*, we performed a 3×NFAT-luciferase reporter gene assay with wild type full length RSK2 and the K216A mutant. The 3×NFAT-luciferase activity was increased in a dose-dependent manner by the presence of wild type RSK2 as was previously reported [13]. However, the RSK2 K216A mutant exhibited impaired activity as compared with wild type at all different concentrations (Figure 8). The *ex vivo* mutation data are consistent with the reduced activity observed in the K216A mutant *in vitro*.

### Implications of NTD RSK2 Structural Diversity

A set of four structural modulations classifies a protein kinase in an “inactive” conformation [38], [39]. The modulations include the distortion of the glycine-rich loop, blockage of the ATP binding site, and activation segment conformation and displacement of the αC-helix. The first two modulations were observed in the NTD MSK1, as described above, and resulted in protein inactivity. A recent clustering of public protein kinase structures based on the conformations of two structural elements, the activation segment (DFG-motif) and the αC-helix, revealed three discrete clusters [40]. Two clusters included inactive kinases with a DFG-out conformation and an αC-helix-out (displaced) conformation. Structures with displaced αC-helices account for 17% of the structures available. A key characteristic of the active conformation of most known kinases is a strong ionic interaction between a positively charged lysine from the β3-strand and a negatively charged glutamic acid from the αC-helix [34], [38]–[40]. By linking the αC-helix to the β3-strand, the lysine-glutamate salt bridge also helps to stabilize the overall fold of the N-terminal lobe [41]. The appearance of the βB-sheet insert in the N-lobe of the NTD RSK2 resulted in the displacement of the αC-helix and disruption of the Lys-Glu interaction, but, nevertheless, the protein exhibited enzyme activity in solution. We present a kinase structure that would be classified as having an “inactive” conformation (the displaced αC-helix), but is actually an active phosphorylated kinase confirmed by complementary protein activity assay.

The αC-helix anchored by a conserved salt bridge toward the N-lobe is known as an important mediator of conformational changes in some protein kinases of the CDK and Src families. They are activated by the movement of the αC-helix from a “displaced” to “active” conformation [42], [43]. In the absence of the activator cyclin, the αC-helix of the CDK2 is rotated outward and the lysine-glutamate ionic pair is disrupted, albeit ATP is bound in the active site [44]. Cyclin binds directly to the αC-helix promoting its inner rotation and restoring the Lys-Glu interaction [45], [46]. The intramolecular interactions of the Src SH3 or SH2 domain with its kinase domain promote the displacement of the αC-helix and disruption of the lysine-glutamic acid ionic pair [43], [47]. Ligand binding to SH3 and SH2 domains activates Src family kinases allowing re-positioning of the αC-helix to the active conformation [48]–[50]. The available crystal structures of other protein kinases in both the inactive and active states showed that the αC-helix is not always the key regulator of activity. For example, the “αC-helix displacement” is not an element of focal adhesion kinase (FAK) regulation because the position of the αC-helix is not changed upon activation [51]. Moreover, the proposed structural coupling between the αC-helix and the T-activation loop does not always occur. The integrity of the αC-helix of PDK1 is not regulated by the T-activation loop phosphorylation [52].

Displaced αC-helix-out conformation in the crystal structure of NTD RSK2 is not equated with lack of protein activity. We believe that the βB-sheet insert compensates for the αC-helix displacement by playing a stabilization role and directing the Lys216 residue toward the active site. Others have reported [23], that upon enzyme activation, the βB-insert of the NTD MSK1 undergoes a structural transition resulting in the appearance of the αB-helix. For this conformational rearrangement, a stable βB-sheet composed of a triad of β-strands should be disrupted first, the two βB-strands would swing apart, and the middle βB-strand must be unfolded and refolded to form a novel αB-helix. Phosphorylation of the T-activation loop in our crystallized NTD RSK2 fragment did not induce those supposed structural rearrangements. However, we cannot exclude the possibility of the βB-sheet unfolding and restoring the αC-helix position and the Lys-Glu ionic interaction *in vivo*. The bacterially expressed E118A NTD mutant was not phosphorylated at Ser227 (Figure S1) and was not active. Whether the activity loss was the result of non-phosphorylation or the mutation of the Glu118 residue is not clear, and we conclude that Glu118 still might be an important mediator of the re-arranged conformation.

The role of the βB-sheet insert is fascinating and needs to be elucidated. This type of insertion in the N-terminal lobe kinase scaffold has never been observed in the kinase domains of other single protein kinases. We believe that this might be a unique feature of two particular kinases, RSK and MSK, which are dual kinases that have two protein kinase domains in a single polypeptide chain. We suggest that the insert might be involved in the correct scaffolding of the full length protein, and might serve as an intermediate structural element connecting the N-terminal kinase domain with the linker region and/or the C-terminal kinase domain. An unusual insert might also be a structural element that is required for synergistic protein activation in a uniquely different regulatory mechanism of dual kinases. The crystal structure of the full length RSK2 or MSK1 might provide insights as to whether the βB-sheet undergoes structural rearrangement and its role in the full length protein.

### The N-Terminal Lobe Groove

**Figure 9:**
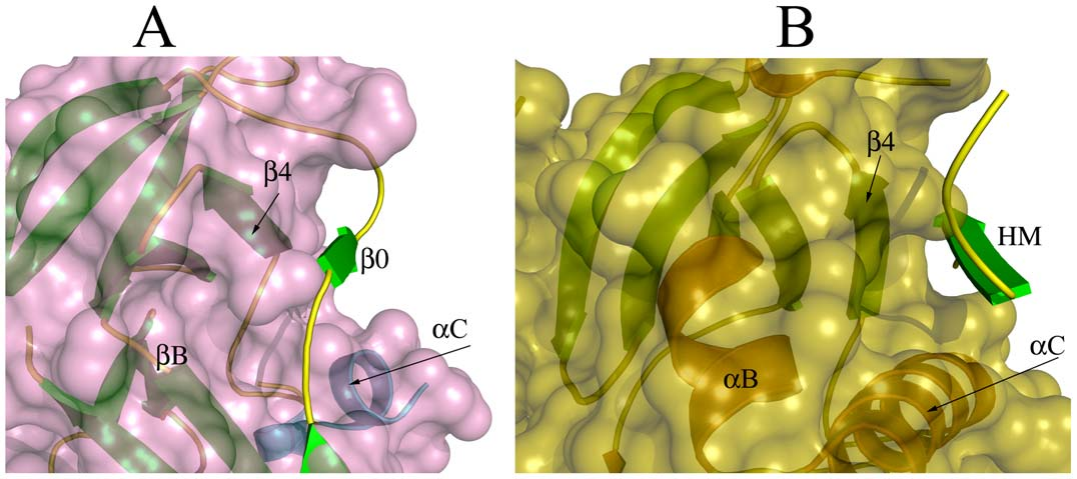
The N-terminal groove. The groove formed in the N-terminal lobe is shown under a semi-transparent surface representation for RSK2 (**A**, pink) and for PKB (**B**, yellow). (**A**) The deep groove in the N-lobe of RSK2 is formed between the β4-strand and the αC-helix and is occupied by the N-terminal β0-strand. Secondary elements of the NTD RSK2 are shown in green and blue. (**B**) The N-lobe pocket in PKB is formed between the β4-strand and the αC-helix. Secondary elements of the PKB (PDB code 1O6K) are shown in green and yellow. The C-terminal hydrophobic motif (HM) is located in the groove on the N-terminal lobe.

The N-terminal lobe has a deep groove on the surface between the αC-helix and the β4-strand junction with the βB-sheet (Figure 9A). Even though the αC-helix is partially disordered in the crystal structure, the groove is clearly defined. We superimposed the structure of the NTD RSK2 with PKB and found that the N-lobe groove is similar to that of PKB (Figure 9B). The groove is known as a hydrophobic motif-pocket for AGC kinases. The N-lobe groove of NTD RSK2 is lined with more hydrophobic residues compared with PKB. Whereas in PKB this region is occupied by the C-terminal hydrophobic motif (HM), in the NTD RSK2, the N-terminal β0-strand (residues 55–57) is anchored to that groove by several hydrogen bonds with the β4-strand. Superimposition of two structures showed that the position of the β0-strand of the NTD RSK2 and the HM of PKB are perfectly overlapped.

The αC-helix, which forms one side of the N-lobe pocket, is not completely ordered in the current structure, and we suggest that the groove might have a different depth due to the ordering of the αC-helix. The N-terminal end appears to play a stabilization role for the isolated NTD RSK2 fragment, which did not have the hydrophobic motif C-terminal end. Similar to our structure, the N-terminal tail occupied a comparable hydrophobic motif groove in the N-lobe in the recently published structure of serum and glucocorticoid-regulated kinase 1 (SGK1), which is also an AGC kinase [53]. Whether the N-terminal end of the full length protein will flip away from the groove allowing the acquisition of the hydrophobic motif linker region, or whether the phosphorylated HM will form the β-sheet with the β0-strand is unknown. The βB-sheet structure might possibly be disrupted if the HM-region displaces the β0-strand that is adjacent to the β1-strand, which contributes in the novel βB-insert.

## Materials and Methods

### Protein Purification and Crystallization

Eighteen N-terminal kinase domain (NTD) fragments of different lengths of mouse RSK2 were cloned into *E.coli* using a full length cDNA clone. Most of the fragments with high-level expression were purified, but failed to crystallize. Longer constructs including the hydrophobic linker region had low solubility and poor yield. Only one construct of the NTD (residues 44–367) produced crystals. The protein was cloned into *NdeI/HindIII* restriction sites of the pET-28a vector (Novagen). The recombinant His-fusion NTD RSK2 was expressed in *E.coli* BL21-Codon Plus (DE3)-RIPL competent cells (Stratagene). The cells were harvested after an additional 5 h of growth at 25°C following induction with 0.2 mM isopropyl-beta-D-thiogalactopyranoside. The cell pellet was resuspended in 30 ml of washing buffer (30 mM imidazole, 500 mM NaCl, 50 mM NaH_2_PO_4_, pH 8.0, 10% glycerol, 10 mM β-mercaptoethanol). Cells were disrupted by French Press (Thermo Electron Co.) and clarified by centrifugation. Soluble His-tagged NTD RSK2 was eluted from nickel-nitrilotriacetic acid agarose (Qiagen) with buffer that contained increasing concentrations of imidazole (100 mM and 200 mM), 150 mM NaCl, and 20 mM Tris pH 8.0. All eluted fractions were combined and diluted in the same buffer without imidazole to prevent protein precipitation. The protein was purified further by size exclusion chromatography on a HiLoad 16/60 Superdex-200 column (GE Healthcare) equilibrated with buffer (150 mM NaCl, 20 mM Tris pH 8.0). We were able to concentrate the protein only when the non-hydrolyzable ATP analogue, adenosine 5′-(β,γ-imido)triphosphate tetralithium salt hydrate, AMP-PNP (Sigma-Aldrich), was added to the protein solution during the concentration step. The final protein stock solution at a concentration of 10–15 mg/ml contained 2 mM AMP-PNP and 10 mM β-mercaptoethanol. The diluted protein at different concentrations (2.5 mg/ml–10 mg/ml) was mixed at a 2∶1 ratio with precipitant solution (6%–10% PEG 3350, 0.2 M proline, 0.1 M Hepes pH 7.5). The crystals were grown at 20°C by the sitting drop vapor diffusion method within 1–2 days. The mutant proteins, K216A and E118A, also had high expression yield and were purified by similar procedures.

### Data Collection and Structure Determination

The crystals were flash-cooled in liquid nitrogen after a short soaking in cryo-protection solution (15% PEG 3350, 0.2 M proline, 0.1 M Hepes pH 7.5, 20% glycerol). The high-resolution data sets were collected at the Advanced Photon Source (APS) microdiffraction beamline 24ID-E using a 20 micron beam. The full data set was collected from three portions of the elongated rod-like crystal. The crystal-to-Quantum 315 CCD detector distance was 250 mm and the crystal was rotated around the spindle axis with images collected over 160° to a resolution of 1.7–1.8 Å. Data were integrated and scaled using the HKL2000 package [54]. The real resolution of the data, used for structure refinement, was estimated taking into consideration the completeness of the last resolution shell, I/σ ratio and R-merge values.

RSK2 crystals belong to the primitive orthorhombic lattice with one molecule in the asymmetric unit cell. The atomic coordinates of the refined MSK1 structure (PDB code 1VZO) were used for the initial crystallographic phasing by molecular replacement. All calculations were performed using PHENIX [55]. With the model given by molecular replacement, a rigid body refinement was carried out at 3.5 Å resolution. All data with a high-resolution limit of 1.8 Å were used for structure refinement. Once a satisfactory description of the protein electron density was complete, water molecules and AMP-PNP were added. A few cycles of slow-cooling annealing (5000→100 K), positional and restrained isotropic temperature factor refinements were followed by visual inspection of the electron density maps, including omit maps, coupled with manual model building (when necessary) using the graphics program COOT [56]. The refined electron density clearly matched the amino acid sequence of RSK2 with the exception of the N- and C-termini (residues 44–46 and 347–367) and two disordered loops 111–119 and 220–230. Strong stereochemical restraints were imposed during the crystallographic refinement and the final RSK2 structure possessed a very good stereochemistry. The r.m.s.d. between two molecules before and after the final round of refinement was less than 0.05 Å. The quality of the stereochemistry of the final protein structure was assessed with the PROCHECK package [57]. The Ramachandran plot showed no residues in generously allowed or disallowed regions (data not shown). As a better guide to the quality of the structure, the values of the free R-factor were monitored during the course of the crystallographic refinement. The final value of free R-factors did not exceed the overall R-factor by more than 5%. R_p.i.m_, precision-indicating merging R factor, was calculated as described [58]. Structural figures and graphical rendering were made using PYMOL (http://pymol.sourceforge.net). The volume calculations were performed with the program CASTp [59].

### In Vitro [γ-^32^P]ATP Kinase Assay

For an *in vitro* kinase assay, the recombinant His-fusion NTD RSK2 (residues 44–367) wildtype and K216A mutant that were purified on a HiLoad 16/60 Superdex-200 column at a protein concentration of 0.5–0.7 mg/ml without the addition of AMP-PNP. A biotinylated S6 peptide, biotin-AKRRRLSSLRA (AnaSpec) was used as a substrate. One µg of NTD (wildtype or K216A mutant) was mixed with 5 µg biotinylated S6 peptide, 1 µCi [γ-^32^P]ATP, 100 µM ATP in kinase buffer, and incubated for 60 min at 30°C. Total reaction volume was 25 µl. Kinase buffer (Cell Signaling) included 25 mM Tris pH 7.5, 5 mM β-glycerophosphate, 2 mM dithiothreitol, 0.1 mM Na_3_VO_4_, and 10 mM MgCl_2_. To show activation with PDK1, the wildtype NTD RSK2 and K216A mutant were pre-incubated with 0.1 µg active PDK1 (Millipore) for 10 min at 30°C in the presence of the ATP mixture. Reaction aliquots (25 µl) were taken as duplicates from the tubes at different time points and added to 7.5 M guanidine hydrochloride (12.5 µl) to terminate the kinase reaction. All required negative controls were performed. Reaction mixture (20 µl) was applied to a SAM Biotin Capture Membrane (Promega Corp.). Membrane washing was performed according to the manufacturer's protocol with 2 M NaCl and 2 M NaCl/1.0% phosphoric acid solutions. The results were analyzed by scintillation counter. Kinetics experiments with the wild type NTD and the K216A mutant were performed under similar conditions.

### Luminescent Assay

The Kinase-Glo^®^ Luminescent Assay (Promega Corp.) was performed in a solid white, 96-well plate in 50 µl reaction according to the manufacturer's instructions. The kinase reaction conditions were optimized with respect to the amount of ATP and kinase substrate as recommended. Serial two-fold dilutions of the NTD RSK2, wildtype or K216A (starting from 10 µg), were made across the plate in kinase reaction buffer (40 mM Tris pH 7.5, 20 mM MgCl_2_, 0.1 mg/ml BSA) containing 0.1 µM ATP and 25 µM S6 peptide (AKRRRLSSLRA) (AnaSpec, San Jose, CA). The kinase reaction was carried out for 60 minutes at room temperature and completed by adding 50 µl of Kinase- Glo^®^ Reagent. The luminescence signal, which is inversely correlated with the amount of kinase activity, was recorded on the Luminoscan Ascent plate reader at 10 minutes after the addition of the Kinase- Glo^®^ Reagent. Effective concentrations (EC_50_ values) were calculated using SigmaPlot2000 software.

### Ex Vivo NFAT3 Activity Assay

The assay was performed as previously described [13]. HEK 293 cells (2.0×10^4^) were seeded into 48-well plates and incubated with 10% FBS-DMEM for 24 h before transfection. The 3×NFAT-luciferase reporter plasmid was transfected with pcDNA3-Flag-NFAT3, pcDNA4-RSK2 (full length), or the pcDNA4-RSK2 K216A mutant (full length). The cells were disrupted after 36 h by the addition of lysis buffer (0.1 M potassium phosphate buffer pH 7.8, 2 mM EDTA, 1 mM DTT, 1% Triton X-100) and analyzed for firefly luciferase activity using the Luminoskan Ascent plate reader. The 3×NFAT-luc luciferase activity was normalized against *Renilla* luciferase activity (pRL-SV40). The experiments were performed as triplicates and reproduced twice. To confirm equal transfection efficiency for the wt RSK2 and mutant, we proportionally increased the amount of protein (200, 400, and 800 ng) and performed Western blot analysis.

### Western Blotting

Phospho Ser227 antibodies (Cell Signaling) were used with purified proteins and the crystal. The crystal was washed three times in the well solution and put in SDS-buffer. The Xpress antibodies (Invitrogen) were used to visualize RSK2 in HEK293 cells.

### Mutagenesis

Point mutations of RSK2 in pET-28 and pcDNA4 vectors were performed using a QuickChange^®^ Lightning site-directed mutagenesis kit (Stratagene) following the recommended protocol, and confirmed by DNA sequencing.

### Accession Numbers

The refined coordinates of NTD RSK2 and structural factor data have been deposited in the Protein Data Bank with the access code 3G51.

## Supporting Information

Figure S1 Western blot shows the phosphorylation of the Ser227 residue in purified proteins (wt NTD, K216A, 50 ng), crystallized wt NTD, and the absence of phosphorylated Ser227 in the E118A mutant. Active full length RSK2 (Millipore) was used as a positive control.
(0.05 MB TIF)

Figure S2 A general cartoon showing the important interactions of amino acid residues with AMP-PNP in the active site, as generated by *Ligplot*.
(1.51 MB TIF)

Figure S3 Western blot confirms an equal level of ectopically expressed proteins - wild type full length RSK2 and K216A mutant in HEK 293 cells after transfection with 200, 400, or 800 ng. This assay is a complement to the luciferase assay presented in Figure 8.
(0.07 MB TIF)
