# Correction: Structural Diversity of the Active N-Terminal Kinase Domain of p90 Ribosomal S6 Kinase 2

**DOI:** 10.1371/annotation/d71909ae-13e2-4fd5-8b17-ca0f13bc38ed

**Published:** 2009-12-22

**Authors:** Margarita Malakhova, Igor Kurinov, Kangdong Liu, Duo Zheng, Igor D'Angelo, Jung-Hyun Shim, Valerie Steinman, Ann M. Bode, Zigang Dong

The Acknowledgments section was omitted. It should read: A part of the work is based upon research conducted at the Northeastern Collaborative Access Team beamline 24ID of the Advanced Photon Source, supported by award RR-15301 from the National Center for Research Resources at the U.S. National Institutes of Health. Use of the Advanced Photon Source is supported by the U.S. Department of Energy, Office of Basic Energy Sciences, under contract No. W-31-109-ENG-38. 

