# Supplementary figures and images for: Structural Diversity of the Active N-Terminal Kinase Domain of p90 Ribosomal S6 Kinase 2
Source: PLoS One. 2009 Nov 30;4(11):e8044. doi: 10.1371/journal.pone.0008044 (PMC2779450; doi:10.1371/journal.pone.0008044)

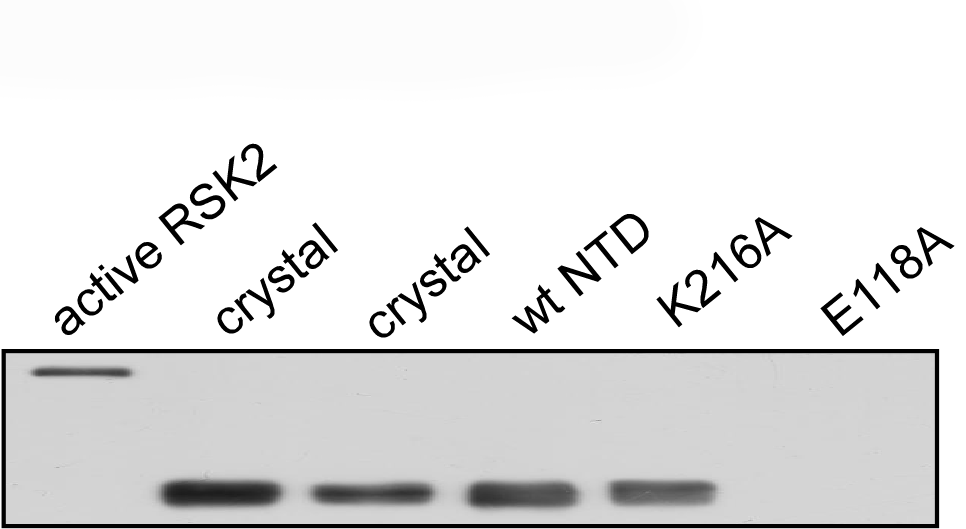

Supplement: Figure S1 — Western blot shows the phosphorylation of the Ser227 residue in purified proteins (wt NTD, K216A, 50 ng), crystallized wt NTD, and the absence of phosphorylated Ser227 in the E118A mutant. Active full length RSK2 (Millipore) was used as a positive control. (0.05 MB TIF) [file pone.0008044.s001.tif]

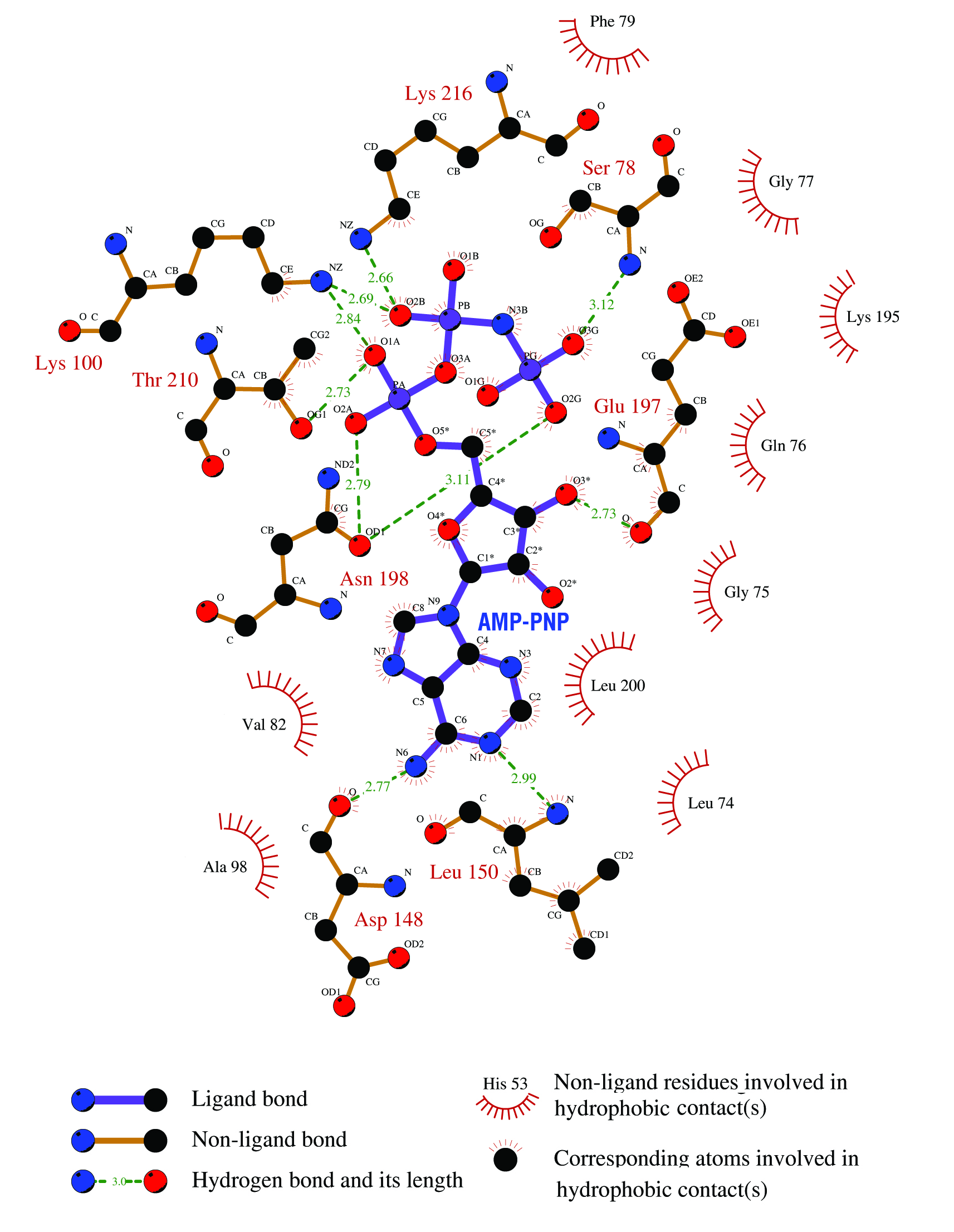

Supplement: Figure S2 — A general cartoon showing the important interactions of amino acid residues with AMP-PNP in the active site, as generated by Ligplot. (1.51 MB TIF) [file pone.0008044.s002.tif]

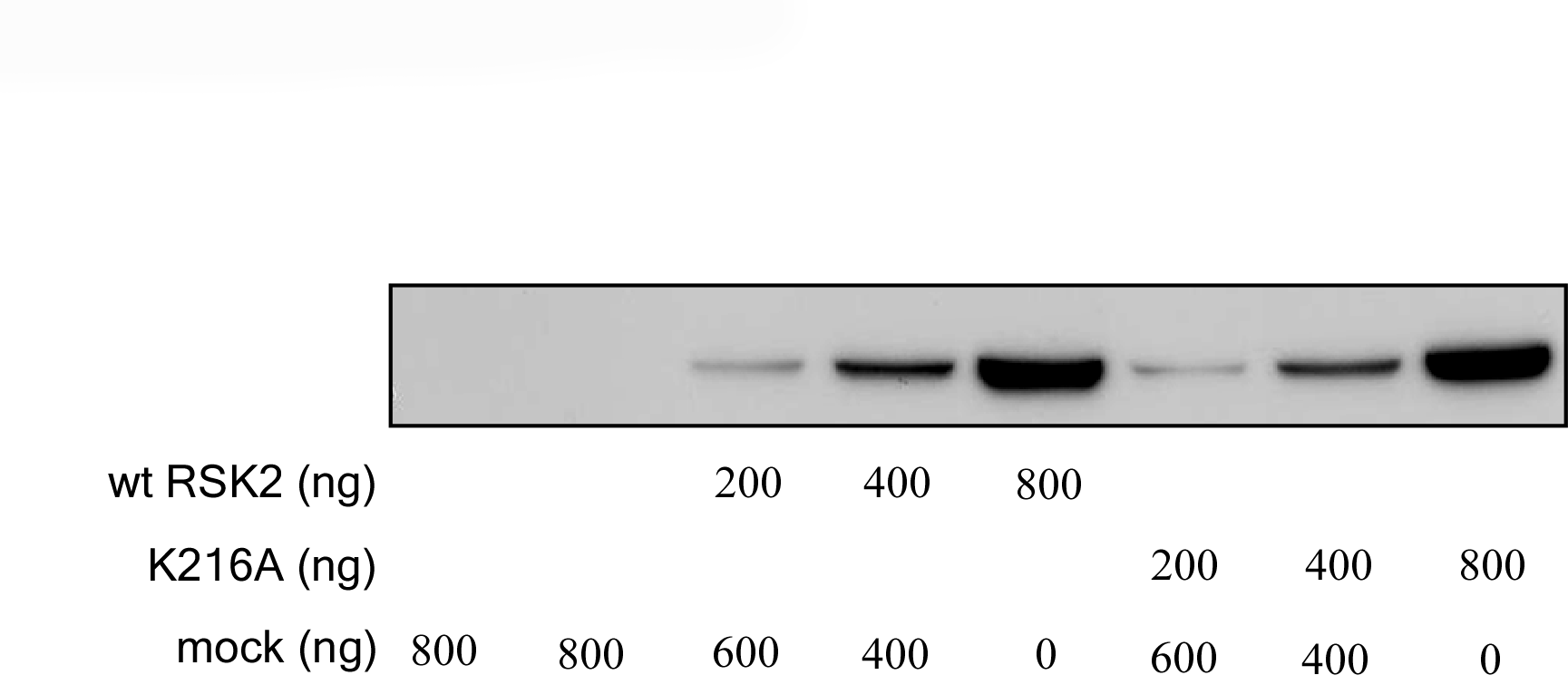

Supplement: Figure S3 — Western blot confirms an equal level of ectopically expressed proteins - wild type full length RSK2 and K216A mutant in HEK 293 cells after transfection with 200, 400, or 800 ng. This assay is a complement to the luciferase assay presented in Figure 8. (0.07 MB TIF) [file pone.0008044.s003.tif]
